# Epidemiology of Injuries in Amateur Male Soccer Players: A Prospective One-Year Study

**DOI:** 10.3390/healthcare11030352

**Published:** 2023-01-25

**Authors:** Afxentios Kekelekis, Zoe Kounali, Nikolaos Kofotolis, Filipe Manuel Clemente, Eleftherios Kellis

**Affiliations:** 1 Laboratory of Neuromechanics, Department of Physical Education and Sport Sciences at Serres, Aristotle University of Thessaloniki, 62100 Serres, Greece; 2 Sport Injury Clinic for Prevention & Rehabilitation, 72100 Aghios Nicolaos, Greece; 3 Escola Superior Desporto e Lazer, Instituto Politécnico de Viana do Castelo, Rua Escola Industrial e Comercial de Nun’Álvares, 4900-347 Viana do Castelo, Portugal; 4 Instituto de Telecomunicações, Delegação da Covilhã, 1049-001 Lisboa, Portugal

**Keywords:** football, football injuries, incidence, amateur players, epidemiology

## Abstract

The purpose of this study was to prospectively monitor and analyze injuries in Greek amateur male soccer players over one competitive season. One hundred and thirty male soccer players in a regional amateur league participated in this study. Injury data and exposure were collected from six teams during training and competition match over one season (2018/19). Injuries were collected weekly and were classified by setting, mechanism, severity, type, calendar distribution, period of injury occurrence, and anatomical location. A total of 103 injuries were recorded during the season, with an incident rate (IR) of 5.5 injuries/1000 h with 95% confidence intervals (CI) values of 4.45 (lower limit) and 6.09 (upper limit). Furthermore, IR was greater for the posterior thigh (IR 1.83/1000 h, 95% CI 1.21–2.44) and hip/groin complex (IR 1.45/1000 h, 95% CI 0.90–1.99) compared to other anatomical locations. Similarly, muscle injuries had greater IR (IR 3.61/1000 h, 95% CI 2.74–4.47) than other tissues. Amateur soccer players had a seven-fold greater chance of getting injured during games (IR 20.76/1000 h, 95% CI 15.28–26.24) rather than during training (IR 3.077/1000 h, 95% CI 2.16–3.80), while injury rates were higher towards the end of a session and peaked in October and February of the season. Based on these results, amateur soccer may benefit from injury prevention strategies incorporated into their regular training practice and focus on muscle injuries, especially in the posterior thigh and the hip/groin complex.

## 1. Introduction

Injuries in soccer negatively affect the team’s performance [1] and the player’s quality of life [2] remaining a severe threat to the player’s career. [3] A plethora of research has focused on injuries in professional soccer [4], but millions of athletes play soccer at a non-professional level. Hence, their participation may also be threatened by injuries.

Our expertise regarding injury rates (IR) in male amateur soccer players remains limited [5,6,7,8,9,10] Injury displays a relatively high range, with values starting from 2.72 injuries [10] of total exposure and reaching 36.9 injuries per 1000 h [9]. These variable IR values indicate that incident rates may vary from study to study, probably due to methodological differences and the sample type examined.

Significant knowledge resulting from injury screening is the anatomical location of the identified injury the type of tissue that sustains an injury, the severity, and the pattern mechanism. Previous research on amateur soccer players has indicated lower limbs to be the most frequently injured body location, including the posterior thigh, the knee, the ankle, and the hip/groin complex [6,11]. The vast majority of injuries were lower limb acute traumatic incidents [5,6,8] involving the muscular tissues [12] and the ligaments [11] while severe bone injuries or concussion incidents were rarely registered. Injury time loss varied between studies ranging from 1 day to over 400 days [5,6,7]. 

High reinjury rates of between 14% and 33% have been reported [6,13], especially for muscle injuries [12,13], ankle ligament injuries [11], and tendinopathies of the lower limb [14]. A higher frequency of recurrent injuries may indicate the need for better management of first-time injury incidents. Due to the limited human and financial resources, injury management in amateur soccer is not the same as in professional soccer. Hence, it is essential that clinicians or coaching staff who work in amateur soccer develop strategies to reduce recurrent injury incidents.

Injury characteristics in amateur soccer have been clearly explained in the literature, pointing out the higher frequency of injury during match play compared to training [9] the in-season injury calendar variation [5], and the trend to occur during the second half of the game than during the first half [15]. If injuries occur during a particular period during the competitive season, then appropriate injury prevention and training management strategies might be necessary. Similarly, the occurrence of injuries during the late phase of a game or training session may highlight the need for better monitoring of athletes’ physical conditioning and training loading, which could then be used to adjust the characteristics of regular training programs.

The literature on amateur soccer injury characteristics is limited, and it is unknown if injury data obtained from professional soccer players can be used to guide injury prevention and management in amateur soccer. Most of the previous studies followed different methodological designs, reporting the frequency distribution of injuries per anatomic location or per tissue [8]. Therefore, data cannot be easily compared between studies, resulting in inconsistency. This can be accomplished by expressing injuries relative to the exposure time. The purpose of this study was to prospectively monitor and analyze injuries in Greek amateur male soccer over one competitive season. We hypothesized that the incidence rate would differ between different anatomical locations and between different tissues. Further, we expected a greater incidence rate during games compared to training. We hypothesized that injury rates would vary during the season and would be greater during the final periods of a game or training.

## 2. Materials and Methods

### 2.1. Study Design

This prospective cohort design followed the STROBE (Strengthening the Reporting of Observational Studies in Epidemiology) statement guidelines [16]. Injury epidemiology and exposure data were obtained weekly for one full season from teams participating in a local amateur league of the Greek football association over the season 2018/2019. The University Ethics Committee approved the experimental protocol. All participants, the medical staff, and team coaches were informed about the research protocol, its benefits, and potential risks. The participants’ written consent was obtained before the start of the study.

### 2.2. Procedures

This study followed a convenience sampling strategy. During the off-season period (June to August), 253 male players from 11 teams participating in a local amateur league of the Greek football association were contacted. Of these, 176 players initially agreed to participate. Forty-six players were excluded before the study since their exposure was not limited to the team’s training sessions and games or could not follow the data collection procedures. 

All players of the teams were eligible for inclusion in this study. Additionally, players that were injured before the beginning of the study and were following a rehabilitation program were included in the study, but that injury was not included in the results. Only injuries sustained during training sessions and games were recorded. Also, players that left their teams were included based on the period they were members of the team. Similarly, players whose injury and exposure data were not monitored in full were excluded from the study. All teams used artificial turf throughout the season for official games and training sessions.

Before the beginning of the monitoring period, the anthropometric characteristics (Age, stature, and body mass) were collected, and body mass index (BMI) was calculated. Players’ preferred leg(s), years of participation, and most frequent playing position were recorded.

The injury data collection procedure followed the international consensus statement for soccer injury surveillance [17]. First, detailed explanations of the methods regarding injury data collection and recording were provided to the medical and coaching staff of the participants’ teams through personal interviews. We particularly explained the importance of accurate diagnosis, when the incidence occurred, which tissues were injured, what was the injury mechanism, and when the player returned to play. Each team member (an assistant coach or physiotherapist) agreed to collect and record exposure data from training and games. They were provided specific instructions regarding data collection procedures. The injury and exposure data were collected every week, then verified and subsequently collected by an author who visited each club weekly.

The official medical physician of each club was responsible for the injury or illness diagnosis, or rarely, the diagnosis was made by the official outpatient department of the local hospital. To achieve consistency, a time-loss injury was described as any absence of the player from a game or training due to any physical cause which resulted from training or matches. The severity level was based on the number of days lost from the initial day of injury to the date of return to full participation and availability for match selection [17]. Therefore, injuries were classified as minimal (1–3 days), mild (4–7 days), moderate (8–28 days), and severe (more than 28 days). Injuries of the same type, which occurred at the same body location as a previous injury within the season, were defined as recurrent injuries [17].

Further, we also recorded the time when the injury occurred during a match or training. A match or training session was divided into three equal periods. For games, there were three periods: the early period (0–30 min), the middle period (31–60 min), and the late period (61 min–end of the match). Similarly, every training session was divided into three equal periods. Training exposure was defined as any players’ physical activity under the guidance of coaching or fitness staff. In contrast, match exposure was defined as a competitive game between different clubs [17]. The calculation of the exposure time was the number of participants multiplied by the total training hours a day. Each player’s attendance was recorded individually. Absence from training or games due to illness or other circumstances was not recorded.

### 2.3. Data Analysis

Data were analyzed using the Statistical Package for MacOS version 27 (SPSS Inc. Chicago, Illinois). Significant differences in each variable between the recorded values and its expected distribution at *p* < 0.05 were examined using Chi-square (x^2^) tests. Particularly, chi-squared tests were used to determine the associations of injuries between anatomic locations, between match play and practice, between different months within the season, and between different timelines during training and/or match play. The effect size (phi) for Chi-square comparisons was also calculated [18]. The level of significance was set at *p* < 0.05. Incidence of injury with 95% confidence intervals (CI’s) was calculated as the number of injuries per 1000 hours of exposure [19].

## 3. Results

**Figure 1:**
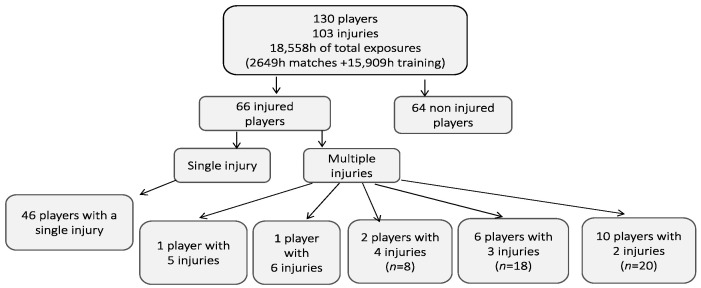
The flow diagram presents the number of players and injuries for one season.

The results from the data collection process are visualized in Figure 1. Of the 152 players that were initially recruited, a total of 130 finished the study (age 21.32 ± 7.42 years; 1.77 ± 0/06 m; body mass 71.23 ± 9.98; BMI = 22.63 ± 2.23). Of these, 66 players reported an injury (56.3% of the sample) with an incidence proportion of 0.507 (95% CI 0.421–0.592). A total of 103 injuries were registered during the season.

### Incidence of Injuries and Characteristics 

The total exposure for all players was 18,558 hours (15,909 training hours and 2649 match hours). The average exposure time was 70 min per training session during the 4-week preseason and the 26-weeks competition period respectively. A typical weekly microcycle consisted of three to four training sessions plus a Saturday match. The overall injury incidence was 5.5 injuries/1000 h (95% CI 4.45–6.04). The recurrent injury rate was relatively low (12 injuries, IR 0.64/1000 h, 95% CI 0.28–1.01 of total exposure). 

Lower limb injuries accounted for 91% of all injuries (94 injuries, 91.26%, IR 5.06, 95% CI 3.88–5.92). Thirty-four injuries occurred at the posterior thigh yielding an IR of 1.83 injuries/1000 h). Other injuries occurred at the hip/groin, knee, and ankle joint (IR 0.59 (Table 1).

Table 2 presents injury distribution per type of tissue. Muscle injuries had the greatest IR followed by ligamentous injuries. There was a statistically significant difference in the frequency of injuries between various tissue types (χ2 = 234.15, df = 6, *p* = 0.0001, phi = 1.50).

The absolute number of injuries did not significantly differ between games and training (χ^2^ =0.476, *p* = 0.490, phi = 0.07). However, the players had approximately 7 times more chances to sustain an injury during games (55 injuries, 53.4%, IR 20.76 injuries/1000 h, 95% CI 15.28–26.24) compared to training sessions (48 injuries, 46.6%, IR 3.017 injuries/1000 h, 95 % CI 2.16–3.8).

Injuries were most commonly mild and minimal, with only four severe injuries sustained during games (Table 3). In total, 1447 days were lost from games and training sessions. The absolute number of injuries significantly differed between severity categories (χ^2^ =30.797, *p* = 0.0001, phi = 0.54).

More than half of the injuries were sustained during the latest third phase of the match or the training session (Table 4). This difference was statistically significant (χ^2^ =24.835, *p* = 0.0001, phi = 0.49).

**Figure 2:**
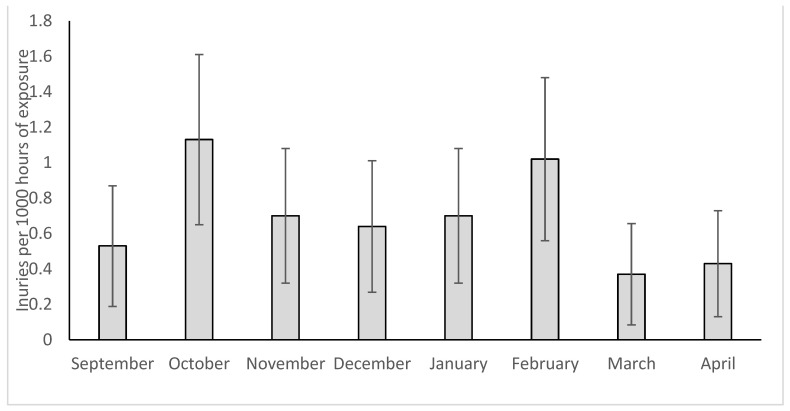
Annual distribution of injuries during training and games. Error bars indicate 95% confidence intervals.

The annual distribution of injuries is presented in Figure 2. Injury frequency differed significantly between various months of the season (χ^2^ =19.471, *p* = 0.007, phi = 0.43). The highest incidence of injuries was reported during October (21 injuries, IR 1.13 injuries/1000 h, 95% CI 0.648–1.61) and February (19 injuries, IR 1.02 injuries/1000 h, 95% CI 0.56–1.48) respectively.

A total of 89 injuries (85%) were non-contact injuries and only 14 injuries were characterized as contact injuries. The number of injuries differed between various injury mechanisms (χ^2^ =103.738, *p* = 0.0001, phi = 1.00). High-speed running and change of direction were reported as the most common mechanisms of injury in this analysis (Table 5).

## 4. Discussion

The results of this study showed that injury IR in one entire season of amateur players was 5.5 injuries/1000 h. Furthermore, IR was greater for the posterior thigh and hip/groin areas than in other anatomical locations. Similarly, muscle injuries had greater IR than other tissues. Amateur soccer players had a seven-fold greater chance of getting injured during games than during training. At the same time, Injury rates were higher towards the end of a session and peaked in October and February of the season.

The overall incidence of injury is in line with some studies [5,13], but it is much lower than the rate reported by van Beijsterveldt et al. (2014) (9.1 injuries/1000 h), or by Kordi et al. (2011) (36.9 injuries/1000 h. The reasons for this marked difference between various studies are unclear. An initial consideration is that there are differences in the type of league and sample characteristics between studies. All studies examined players who participated in regional amateur leagues in their respective countries, with similar participation characteristics (two to three training sessions plus a game a week). Our sample of players was younger than the players examined by van Beijsterveldt et al. (2014) and those examined by Souza et al. (2013). Hence, it is improbable that factors such as the league type or age influenced the differences in the recorded IR between studies. Methodological differences might have contributed to variations in registered injuries between studies. Still, this effect should have been minimal since all of the studies used the same injury and exposure definitions (after Fuller et al.). Since the injury rate which has been reported by van Beijsterveldt et al. (2014) is very similar to rates that have been reported for professional players (as reported in a recent systematic review [4], it can be suggested that training and game intensity level of players that participated in the study by van Beijsterveldt et al. (2014) might have been different (presumably greater) than the teams that participated in our study. It should be mentioned that there may be other factors that influence the IR, such as the workload that is applied to the players [20], the application of injury prevention strategies or not [21], the player’s well-being [22] or the game calendar [23]. The impact of these additional factors on the differences in IR between studies is unclear and subject to further investigation.

Our results revealed that amateur players sustained about 2 posterior thighs and 1.5 hip/groin injuries per 1000 h of participation (Table 1). Similar findings to our study were reported by Sousa et al. (2013); however, in their research, injuries of the hip/groin were relatively low (8%). Posterior thigh injuries mostly refer to hamstring injuries, which are critical injuries for amateur and professional soccer [24]. Similarly, injuries in the hip/groin area can have an important impact on an athlete’s career [25]. Together with knee injuries, these specific types of injuries have a multi-factorial origin, and previous research has shown that there is no single solution for their management and prevention [26,27,28]. In amateur soccer league players, such as the ones examined the present study, financial resources for treating and preventing injuries are limited (relative to professional soccer). In addition, we have observed that amateur players at this level of play were not very knee on participating in specific injury prevention exercises, in addition to their weekly training schedule. Hence, incorporating injury prevention strategies into their weekly training practice may be a first-step measure to reduce injuries at this level of play.

The present results showed that muscle injuries had almost three times greater IR compared to ligamentous injuries and even greater IR than other types of injuries (Table 2). This is in line with previous studies on amateur players [6,8,13]. Even though some studies reported a greater percentage of ankle sprains [10] or joint/ligamentous injuries in total [6,7] than the present study, it is clear that injury prevention strategies which aim to reduce muscle and ligamentous injuries may be particularly beneficial for amateur players.

We observed that match injury incidence was approximately nine times higher than training injury incidence (Table 3). This is in line with previous studies [5,6,8,13], and it can be attributed to the greater play intensity when playing an official game compared to training practice [5]. In addition, in this study, most injuries were characterized as minimal or mild, and only 5% were severe (Table 3). Other studies have reported a greater percentage of severe injuries, varying from 18% to 47.7% [5,7]. These differences between studies may be partly due to differences in the methodology of injury assessment. For example, van Beijsterveldt et al. (2014) used a web-based injury system where players reported their injuries in consultation with paramedic staff. In our study, injuries were recorded on-site by the paramedic or medical staff. Hence, it is expected that variations in the qualitative assessment of injury severity influence the final classification of injuries. 

In our study incidents of head and neck injury were not reported. A great effort has been recently acknowledged to understand the extent and the impact of a brain injury on the player’s quality of life [29,30]. According to previous studies, incidents of concussion were rarely registered in amateur soccer. Nevertheless, based on our findings, the specific cohort of amateur Greek players experienced a mild injury profile, mostly during the games.

The re-injury rate was 12% (0.6 injuries/1000 h) which is almost half of those reported for Dutch (1.3/1000 h) [6], or Swedish (35%) amateur players [13]. The low re-injury rates observed in our analysis were surprising. This is due to the fact that we qualitatively observed that amateur players that play in this league do not have standard and continuous medical and physiotherapy support, at least not the same as the services provided in professional soccer. Further, no injury prevention strategies were systematically applied. Based on the collected data, we can only speculate that since most injuries were mild or moderate, there were fewer re-injuries. Nevertheless, further research is necessary to examine the return-to-play strategies in a larger sample of players at this level.

In agreement with our hypothesis, IR was greater during the last part of the game or training. This result agrees with previous studies [5,15], and it can be attributed to the influence of fatigue during the game or training. Hence, appropriate physical conditioning programs to improve fatigue can benefit amateur players. 

Spikes in injury incidence were observed during October and February (Figure 2). These correspond to periods immediately after the pre-season (October) or Christmas break (February). Sousa et al. (2013) also reported two spikes, one in November and the second in February, corresponding to the present data. The higher IRs during these specific periods could be due to the greater intensity of play [5] and more games in a row than in other season periods. Worsening weather conditions are also observed in February, which might have contributed to the increase in injury frequency. Therefore, these results indicate that for amateur players who follow this specific calendar, special attention should be given to preventing or managing injuries immediately after the preseason and Christmas break periods.

There are some limitations in this study. First, the sample size was relatively small. Injuries were defined as time-loss injuries. Therefore, any players’ problems that required medical assistance but did not result in time loss were not considered. In addition, injuries were not classified as traumatic or overuse due to difficulties in obtaining valid pre-injury medical data from all players. The initial diagnosis in very mild incidents was based on a clinical examination by the medical staff, and, in some cases, the precise tissues that were injured were not identified. Due to the nature of this study, it couldn’t be established internal validity regarding the medical examination and the diagnosis of the injury. Finally, although we recorded player participation by direct contact with the players weekly, this remains an estimate of player exposure.

## 5. Conclusions

In conclusion, amateur soccer players in a regional Greek league suffered mostly from muscle strains and ligamentous injuries of mild or moderate intensity, mainly during games. Injuries increased at the end of sessions and peaked in October and February, while the recurrent injury rate was relatively low. Based on these results, it could be suggested that amateur soccer may benefit significantly from injury prevention strategies incorporated into their regular training practice and focus on muscle injuries, especially in the posterior thigh and hip/groin areas. More attention to preventing injuries should also be paid to the players during periods of the season of continuous games.

## Figures and Tables

**Table 1:** Number of injuries, injury rate (number of injuries per 1000 h of exposure), and 95% confidence intervals (CI) according to anatomical location.

Injury Location	*n*	Injury Rate	95% CI
Posterior thigh	34	1.83	1.21–2.44
Hip/Groin	27	1.45	0.90–1.99
Knee	13	0.7	0.32–1.8
Ankle	11	0.59	0.24–0.94
Anterior thigh	4	0.21	0.004–0.42
Low back	3	0.16	−0.02–0.34
Foot/heel	3	0.16	−0.02–0.34
Lower leg/ankle	2	0.1	−0.042–0.256
Ribs	2	0.1	−0.042–0.256
Elbow	2	0.1	−0.042–0.256
Wrist	2	0.1	−0.042–0.256

**Table 2:** Percentage of injuries, injury rate (number of injuries per 1000 h of exposure), and 95% confidence intervals (CI) according to the type of injury.

Type of Injury	*N* (%)	Injury Rate	95% CI
Muscle Injury	67 (65)	3.61	2.74–4.47
Ligament injury	20 (19.4)	1.07	0.59–1.54
Contusions-bruises	7 (6.8)	0.37	0.098–0.656
Meniscus Injury	2 (1.9)	0.10	−0.042–0.256
Bone injuries	3 (2.9)	0.16	−0.02–0.34
Fractures	1 (1)	0.05	−0.051–0.159
not specified	3 (2.9)	0.16	−0.02–0.34

**Table 3:** Percentage of injuries, injury rate (number of injuries per 1000 h of exposure) and 95% confidence intervals (CI) during games and training according to injury severity.

Injury Severity		Injuries (*n*)%	IR	95% CI
minimal	Games	18	6.79	3.66–9.90
	training	15	0.94	0.465–1.41
	Total	33 (32 %)	1.77	1.16–2.37
Mild	Games	22	8.3	4.83–11.7
	training	20	1.25	0.70–1.80
	Total	42 (40.8%)	2.26	1.57–2.94
moderate	Games	11	4.1	1.7–6.6
	training	13	0.81	0.59–1.04
	Total	24 (23.3%)	1.29	0.77–1.80
Severe	Games	4	1.51	0.04–2.9
	training	0	0	0
	Total	4	0.21	0.004–0.42

**Table 4:** Percentage of injuries, injury rate (number of injuries per 1000 h of exposure) and 95% confidence intervals (CI) occurring in the first (early), second (middle) or final (final) one third of a game or training session.

	*n* (%)	IR	95% CI
Early	20 (19.4)	1.07	0.59–1.54
Middle	25 (24.3)	1.34	0.812–1.86
Late	58 (56.3)	3.12	2.31–3.92

**Table 5:** Percentage of injuries, injury rate (IR, number of injuries per 1000 h of exposure), 95% confidence intervals (CI) for contact and non-contact injuries, and corresponding injury mechanisms.

	*n* (%)	IR	95% CI
non-contact	89 (86.41)	4.79	3.79–5.78
Contact	14 (13.6)	0.74	0.359–1.14
High Speed running	29 (28.2)	1.56	0.99–2.12
Change of Direction	16 (15.5)	0.86	0.44–1.28
Deceleration	8 (7.8)	0.43	0.13–0.729
Landing	7 (6.8)	0.37	0.098–0.656
Kicking	6 (5.8)	0.32	0.065–0.581
Overstretching	5 (4.9)	0.26	0.033–0.50
Fall	5 (4.9)	0.26	0.033–0.50
Acceleration	4 (3.9)	0.21	0.004–0.42
Surface	4 (3.9)	0.21	0.004–0.42
Valgus	2 (1.9)	0.1	−0.042–0.256
Tackling	2 (1.9)	0.1	−0.042–0.256
Passing	1 (1)	0.053	−0.051–0.159

## Data Availability

Not applicable.
